# Status of Tobacco Smoking and Diabetes with Periodontal Disease

**DOI:** 10.31729/jnma.3610

**Published:** 2018-10-31

**Authors:** Sujaya Gupta, Anjana Maharjan, Bhageshwar Dhami, Pratikshya Amgain, Sanjeeta Katwal, Bidhya Adhikari, Ashutosh Shukla

**Affiliations:** 1 Department of Periodontics, Kantipur Dental College, Basundhara, Kathmandu, Nepal; 2 Department of Dentistry, Patan Academy of Health Sciences, Lagankhel, Lalitpur, Nepal; 3 Osho Smile Dental Clinic, Balaju, Kathmandu, Nepal; 4 Subidha Polyclinic, Belbari, Morang, Nepal

**Keywords:** *diabetes*, *pack years*, *periodontitis*, *risk factors*, *smoking*

## Abstract

**Introduction:**

Periodontitis is multifactorial disease that along with dental caries remains one of the commonest cause of tooth loss worldwide. Effective management requires clear understanding of risk factors. Smoking has a dose-dependent effect on periodontium. Similarly, individuals with diabetes have severe forms of periodontal diseases. We aim to assess the prevalence of periodontal disease in dental patients in relation to smoking and diabetes.

**Methods:**

The study was conducted among 522 patients visiting the Periodontics Department, Kantipur Dental College. Individuals willing to participate had to sign an informed consent and undergo interview and clinical examination. Data collection, done on a structured proforma, was analysed using SPSS 20.0.

**Results:**

Prevalence of periodontitis was 372 (71.3%), diabetes 33 (6.3%) and smoking as 138 (26.4%). Hypertension was observed in 64 (12.3%) patients and family history of diabetes among 94 (18%). Among the 372 periodontitis patients, smoking behaviour was present in 120 (32.3%), diabetes in 32 (8.6%), family history of diabetes in 72 (19.4%) and hypertension in 62 (16.7%). Conversely, 120 (87%) smokers, 33 (97%) diabetics, 72 (76.6%) with family history of diabetes, 62 (96.9%) hypertensive, 216 (41.4%) male and 156 (29.9%) female participants had periodontitis. Smoking behaviour was more in males: 115 (39.4%) compared to 23 (10%) females.

**Conclusions:**

Periodontitis was significantly associated with smoking, diabetes, hypertension and age. It is recommended that tobacco cessation and diabetes control be promoted as an integral component of periodontal therapy and oral health be included as an essential element of general health when conducting national health surveys.

## INTRODUCTION

Periodontitis is a chronic inflammatory disease that affects more than 50% of adult population worldwide.^1, 2^ A hospital-based study in Nepal showed that staggering 52.5% suffered from gingivitis and 47.5% from periodontitis.^3^ Along with caries, periodontitis remains the commonest cause of tooth loss. As periodontitis is multifactorial, effective disease management requires a clear understanding of all associated risk factors.

The commonest risk factors attributed to periodontal diseases are tobacco smoking, diabetes, pathogenic bacteria and tooth deposits.^4^ Smoking not only impacts the outcome of non-surgical periodontal therapy but also surgical therapy and long-term success of dental implant placement.^4^ Periodontitis is considered sixth complications of diabetes mellitus (DM).^5^ Individuals with diabetes are more likely to have periodontal disease than without it.^6–9^ Research has for long, suggested a two-way relationship between DM and periodontal diseases.^6, 8^

**In this context** we aim to assess the prevalence of periodontal disease in dental patients in relation to smoking and diabetes

## METHODS

A descriptive cross-sectional study was carried out among 522 patients visiting the department of Periodontics, Kantipur Dental College, Teaching Hospital, Kathmandu, Nepal (KDC) from 2018 March to May.

Ethical clearance was obtained from Institutional Review Committee (KDC-IRC). The individuals were explained the relevant details, aim and procedure of the study by researchers. Those willing to participate had to sign an informed consent and undergo interview and clinical examination. Confidentiality was maintained: No names, documents or results were disclosed or circulated anywhere other than hospital doctors. The names of the participants do not appear in the final report.

The inclusion criteria included i) male and female individuals visiting Periodontics department, ii) presence of periodontal disease (gingivitis or periodontitis) with bleeding on probing (BOP), and/or periodontal probing depth (PPD) > 4 mm or loss of attachment (LOA)>1 mm on at least one tooth (anterior or posterior), and iii) minimum of 16 natural uncrowned teeth. The exclusion criteria were: i) periodontal therapy done within past three months, ii) presence of other systemic diseases or infections besides type 2 DM and hypertension (HTN) that influence the periodontal status like: rheumatoid arthritis, thyroid disorders, epilepsy, inflammatory bowel disease, tuberculosis, pneumococcal pneumonia, etc; iii) lactating, pregnant or intention of becoming pregnant at the time of examination, iv) individuals taking oral contraceptive pills, v) history of systemic antimicrobial administration within last three months, vi) patients requiring emergency management, and vii) refusal of informed consent.

Convenience (non-probability) sampling method was utilised and a sample size of 448 was calculated using data from the study^1^ in following formula:


$$\text{n =}\frac{({\text{Z}}^{2}\times \text{p (1-p))}}{{\text{e}}^{2}}=447.78$$

Where,

n = required sample size
Z = Z value = 1.96 at 95% confidence level
p = expected population proportion; percentage expressed as decimal = 0.248 (24.8%)^1^
e = margin of error = 0.04 (4%)

Data collection was done on a structured proforma developed that included basic demographic information, periodontal status, systemic condition mainly HTN and diabetes and smoking behaviour. Duration of diabetes, medication history and family history were also recorded. In smoking history, number of cigarettes smoked per day, pack-years of smoking and smoking status as current, former or nonsmoker was recorded. “Pack-years” was calculated as follows:


$$\begin{array}{l} \text{Pack Years = (Number of cigarettes smoked per day }\times \\ \text{Number of years smoked)/20} \end{array}$$

Armamentarium utilized were mouth mirror and periodontal probe with William's markings (millimeter calibrations at 1,2,3,5,7,8,9 and 10 mm). Data were entered into Microsoft Excel and analysed using Statistical Package for the Social Sciences (SPSS) for Windows version 20.0. Armonk, NY: IBM Corp., SPSS Statistics. The level of significance was set at 0.05. Pearson's Chi-square test was utilised for categorical attributes and Student's Independent t-test was used for numerical variables.

## RESULTS

The total number of participants was 522. Among them, the prevalence of periodontitis was observed to be 372 (71.3 %), diabetes 33 (6.3%) and tobacco smoking as 138 (26.4%) (Table 1, 2). Hypertension was seen in 64 (12.3 %) patients and family history of diabetes among 94 (18%).

Among the 372 periodontitis patients, the tobacco smoking was observed in 120 (32.3%), diabetes in 32 (8.6%), HTN in 62 (16.7%) with P<0.001 (Table 1).

The association of periodontitis was found to be statistically significant with tobacco smoking, diabetes, HTN and age but not with sex and family history of diabetes (Table 1, 3).

**Table 1.** Relationship of periodontitis with tobacco smoking behaviour, diabetes, family history of diabetes and HTN (Pearson's x^2^ test).

Parameters	Periodontitis n (%)		P
	Yes	No	
Tobacco smoking (N = 138)	120 (87)	18 (13)	<0.001
Diabetes (N = 33)	32 (97)	1 (3)	0.001
Family history of diabetes (N = 94)	72 (76.6)	22 (23.4)	0.207
HTN (N = 64)	62 (96.9)	2 (3.1)	<0.001
Sex			
Male (N = 292)	216 (41.4)	76 (14.6)	0.123
Female (N = 230)	156 (29.9)	74 (14.2)	

Among the 522 participants, 292 (55.9%) were male and 230 (44.1%) were female. They are categorised on the basis of age (Table 2).

**Table 2:** Age*categories in accordance with the prevalence of periodontal disease, diabetes, family history of diabetes, smoking, HTN and sex (N = 522).

Age Category (years)	Periodontal Disease		Diabetes	Family History of Diabetes	Smoking	HTN	Sex		Total n (%)
	Gingivitis n (%)	Periodontitis n (%)					Male n (%)	Female n (%)	
≤ 30	114 (21.8)	98 (18.8)	-	30 (5.7)	51 (9.8)	-	119 (22.8)	93 (17.8)	212 (40.6)
31 - 45	29 (5.6)	142 (27.2)	11 (2.1)	35 (6.7)	30 (5.7)	15 (2.9)	97 (18.6)	74 (14.2)	171 (32.8)
46 - 60	6 (1.1)	92 (17.6)	18 (3.4)	24 (4.6)	36 (6.9)	29 (5.6)	55 (10.5)	43 (8.2)	98 (18.8)
61–75	1 (0.2)	40 (7.7)	4 (0.8)	5 (1)	21 (4)	20 (3.8)	21 (4)	20 (3.8)	41 (7.9)
Total	150 (28.7)	372 (71.3)	33 (6.3)	94 (18)	138 (26.4)	64 (12.3)	292 (55.9)	230 (44.1)	522

* *Minimum=12 years; Maximum=75 years; Mean±S.D. (Years)=36.83±13.92*

**Table 3:** Relationship of periodontitis with age and various parameters of tobacco smoking.

	Periodontitis	Sample (n)	Mean±S.D.	Standard error mean	P
Age (years)	Yes	372	41±13.359	0.693	<0.001
	No	150	26±9.063	0.740	
Years of smoking	Yes	119	14.36±11.303	1.036	<0.001
	No	19	7.15±4.003	0.918	
Cigarettes per day	Yes	119	7.82±7.091	0.650	0.429
	No	19	6.47±5.004	1.148	
Pack years of smoking	Yes	119	6.25±8.594	0.787	<0.001
	No	19	2.45±2.501	0.573	

Independent samples t-test (2-tailed) at confidence interval 95%

**Figure 1.**
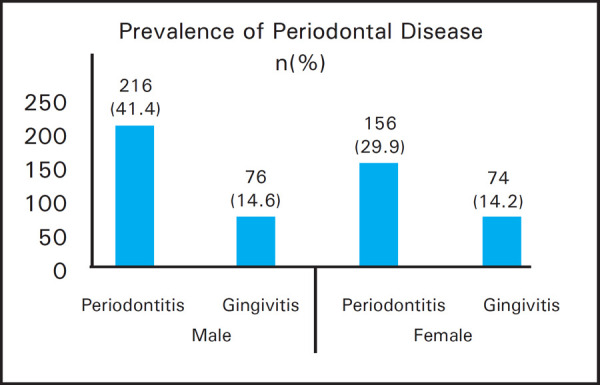
Prevalence of periodontal disease according to sex.

**Figure 2.**
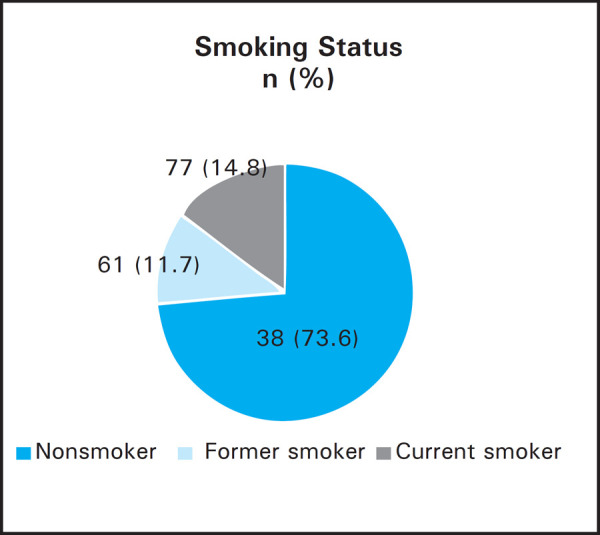
Smoking status of the participants.

The prevalence of periodontal diseases and smoking status has been compared with sex (Table 4, Figure 1) and smoking status of all participants has been presented (Figure 2). The duration of smoking habit was 13.207±10.933 years with standard error mean 0.930.

**Table 4:** Relationship between smoking behaviour and sex.

	Smoking				
		Yes n (%)	No n (%)	Total n (%)	P
Sex	Male	115 (39.4)	177 (60.6)	292 (100)	
	Female	23 (10)	207 (90)	230 (100)	<0.001
	Total	138	384	522	

## DISCUSSION

Periodontal diseases: gingivitis and periodontitis, are collectively the most common diseases known to mankind. They were the most common of all diseases found in the embalmed bodies of the ancient Egyptians.^4^ In gingivitis, inflammation is confined to gingiva, which is reversible with meticulous oral hygiene. Periodontitis on the other hand signifies inflammation that has extended to periodontal ligament and alveolar bone. It is a chronic condition, characterised by the destruction of periodontal tissues that result in LOA of connective tissue and destruction of alveolar bone.^5, 8^ Collagen fibres in periodontal ligament when destroyed, lead to the formation of “periodontal pockets.” Periodontitis is mainly irreversible and slowly progressing disease. Initially it is typically asymptomatic so that the individual is often unaware until the inflammation and destruction of tissues has led to alveolar bone loss, tooth mobility and ultimately tooth loss. The worldwide prevalence of periodontitis is more than 50% of adult population.^1, 2, 8^ Whereas, a hospital-based study in Nepal showed 52.5% suffered from gingivitis and 47.5% from periodontitis (28.3% localized, 18% generalized).^3^ Another hospital-based study reported 68.33% had gingivitis and 40% had periodontitis.^10^ In rural Nepalese population gingival recession was observed in 65.44%. ^11^ In the current study, periodontitis was observed in 71.3%. This was similar to those reported in India,^12–14^ Brazil, and Chile: 62.6% and 58.6% respectively (>5 mm LOA)^15^ but higher than reported in Chinese (25.9%)^2^ and Korean (24.8%)^1^ population (shallow and deep periodontal pockets). The reason for higher prevalence could be that this study was conducted at specialised periodontics clinic where the chances of individuals having periodontitis is always higher than general dental clinic.

To recognise and appreciate the risk factors, it is important to understand the aetiological factors and the pathogenesis of periodontal disease. Periodontal destruction is due to inflammatory host response secondary to infection by periodontal pathogens modified by risk factors.^16, 17^ As periodontal disease is multifactorial, effective disease management requires a clear understanding of all the associated risk factors.^16^ Periodontitis is associated with age, inadequate oral hygiene, smoking, obesity, socioeconomic status and chronic diseases such as cardiovascular disease, osteoporosis, and diabetes.^16, 17^ Among them, the commonest risk factors attributed to periodontal disease are: tobacco smoking, diabetes, pathogenic bacteria and microbial tooth deposits.^4^ Also, periodontal disease is more severe in smoker diabetics than non-smoker diabetics.^18^

Globally more than seven million people are dying each year by tobacco.^19^ Although smokers show less signs of clinical inflammation and gingival bleeding compared to non-smokers,^16,20, 21^ there is strong dose-dependent influence of smoking on periodontal tissues with increased severity in smokers.^4, 21, 22^ Smoking not only impacts the outcome of non-surgical periodontal therapy but also surgical therapy and long term success of dental implant placement.^4, 23, 24^ Tobacco smoking modifies the host response to the challenge of bacteria in microbial dental plaque. The adult smoking prevalence in Nepal is 18.5% (STEPS survey 2012–2013).^19^ In current study, smoking was present in 26.4%, similar to Dhami et al,^21^ Hong et al,^1^ and Han et al.^25^ The higher prevalence in the sample further justifies tobacco smoking as risk factor for periodontitis. Periodontitis was significantly associated with duration of smoking, pack years of smoking but not with cigarettes per day (Table 3). The relationship of smoking behaviour with sex was found to be highly significant (P<0.001) with more male smoking population, similar to other studies.^26^ Majority of the participants were nonsmokers (73.6%). However, this may not present the true picture of smoking status as social and cultural aspects often influence the self-reporting of smoking behaviour and many female smokers might not have accurately responded.^27–28^

DM is another chronic condition that has been associated with periodontitis. Patients with undiagnosed or poorly controlled DM type 1 or type 2 are at higher risk for periodontal disease.^7–16^ Twenty five years ago, Loe had stated periodontitis to be the sixth complications of DM.^5^ LOA and bone loss start early in the diabetic population. Inadequately controlled diabetes cause a severe breakdown of periodontal tissues.^6–9^ Also, the periodontal disease rate in type 2 DM was three times than in nondiabetic individuals.^5–8^ There is sufficient evidence to support the bi-directional relationship between DM and periodontal disease.^5, 8, 29^ Research has long suggested periodontitis to affect the glycaemic control in diabetics and periodontal therapy to improve it.^29–30^ In this study, self-reported diabetes was present in 6.3%.

This study further ascertains the effect of tobacco smoking behaviour, diabetes, hypertension and age over prevalence of periodontal diseases. Total of 87% of smokers and 97% of diabetics had periodontitis similar to previous studies.^1, 21, 25^ The association of periodontitis was found to be statistically significant with tobacco smoking, diabetes, hypertension and age but not with sex and family history of diabetes (Table 1, 3). Higher age predicted greater incidence of periodontal disease, however no difference in the proportion of periodontal disease was seen in male and female similar to previous studies. ^1–5^

The limitations of the study include: only those volunteers coming to KDC were enrolled in the study; other confounding factors and underlying systemic conditions were not taken into consideration; and a longer study period would have included larger population.

## CONCLUSIONS

High prevalence rate of periodontitis and statistically significant association was observed with smoking behaviour and diabetes. However, only a handful of studies have been conducted exploring the numerous risk factors of gingivitis and periodontitis in Nepalese population, and none from the government. Unlike the national level health surveys of both developed as well as developing countries, no such surveys exist for the prevalence of various forms of oral and periodontal diseases in Nepal. Further studies in this context especially from the government sector is highly needed. Hence, it is recommended that oral health be included as an essential element of general health when conducting national level health surveys by the Government of Nepal. Furthermore, healthy systemic condition and good oral behaviour can lead to healthier oral and periodontal status. Thus, tobacco cessation and diabetes control should be prompted as an integral component of periodontal health and therapy.
